# Multisystem Inflammatory Syndrome: A Case in an Adult With Controlled Lymphoma and a Persistent SARS-CoV-2 Infection

**DOI:** 10.7759/cureus.40776

**Published:** 2023-06-22

**Authors:** Luca Pipitò, Alice Medaglia, Irene Russotto, Silvia Bonura, Antonio Cascio

**Affiliations:** 1 Department of Infectious and Tropical Diseases, Azienda Ospedaliera Universitaria Policlinico (AOUP) Paolo Giaccone, Palermo, ITA; 2 Department of Health Promotion, Mother and Child Care, Internal Medicine and Medical Specialties "G. D'Alessandro", University of Palermo, Palermo, ITA

**Keywords:** sars-cov-2 persistence, pericardial effusion, pancytopenia, fever of unknown, multisystem inflammatory syndrome

## Abstract

Multisystem inflammatory syndrome (MIS) is a new and rare complication of COVID-19 that usually occurs in children. An increasing number of cases of MIS in adults are described in the literature. The condition is associated with high mortality, and treatment is non-standardized. Clinical pictures are heterogeneous, and diagnosis is very challenging. Here we describe a case of MIS in a 60-year-old man with previous follicular lymphoma treated with obinutuzumab and recent SARS-CoV-2 infections. He complained of an unknown fever and developed pancytopenia during the hospitalization, associated with a general clinical worsening. The patient was successfully treated with intravenous immunoglobulin and steroids.

## Introduction

Severe acute respiratory syndrome coronavirus 2 (SARS-CoV-2) led to global coronavirus disease 2019 (COVID-19) pandemic. The virus affects the respiratory system predominantly and has resulted in several complications. Multisystem inflammatory syndrome (MIS) is a new and rare complication of COVID-19, which develops 2-12 weeks after the onset of acute COVID‐19. It is characterized by extrapulmonary multiorgan involvement (cardiovascular, respiratory, dermatologic, hematologic, and neurologic systems), and it’s associated with high mortality [1,2]. Children are usually affected, where the disease resembles a Kawasaki-like syndrome [3]. However, an increasing number of cases of MIS in adults (MIS-A) are described in the literature. According to the Centers for Disease Control and Prevention, the MIS-A case definition is based on fever (≥38.0°C), primary and secondary clinical criteria, laboratory evidence of inflammation and recent or current SARS-CoV-2 infection [4]. Treatment of MIS-A remains unclear and non-standardized [5]. Here we reported a case of MIS-A, treated successfully with steroids and intravenous immunoglobulin.

## Case presentation

**Figure 1:**
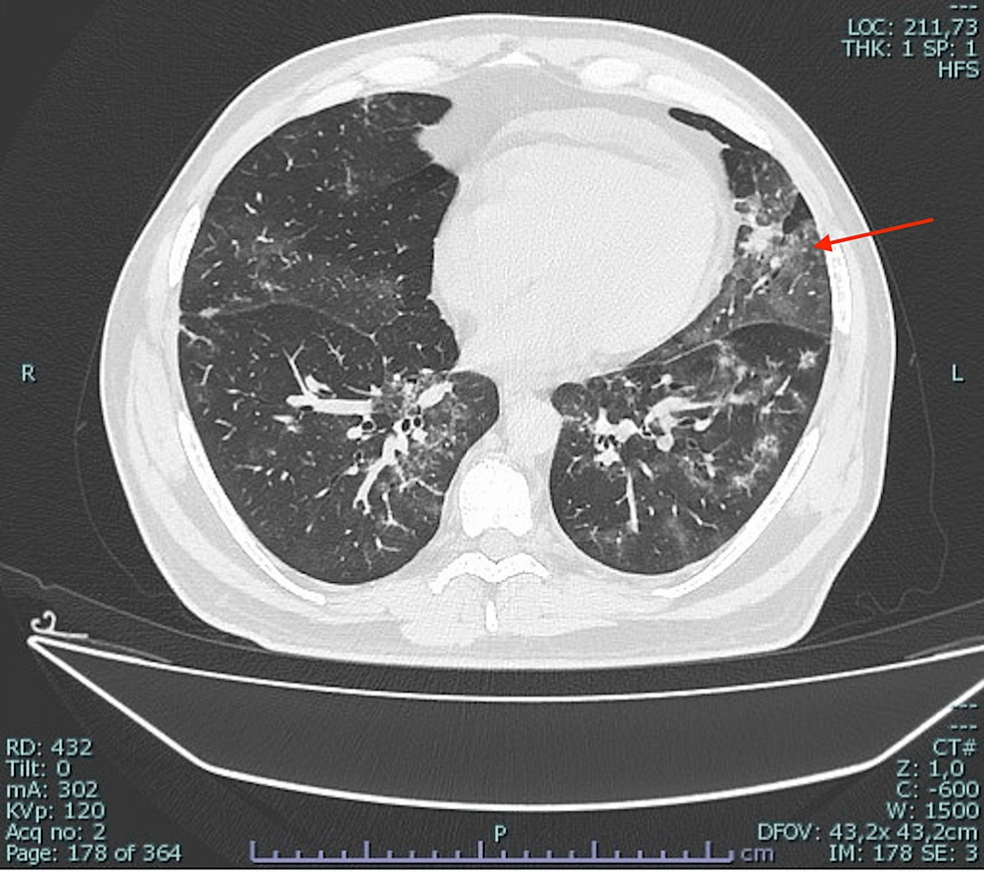
Chest CT shows a bilateral patchy distribution of ground-glass areas associated with consolidation; the arrow indicates a consolidation in the left lung lower lobe.

**Figure 2:**
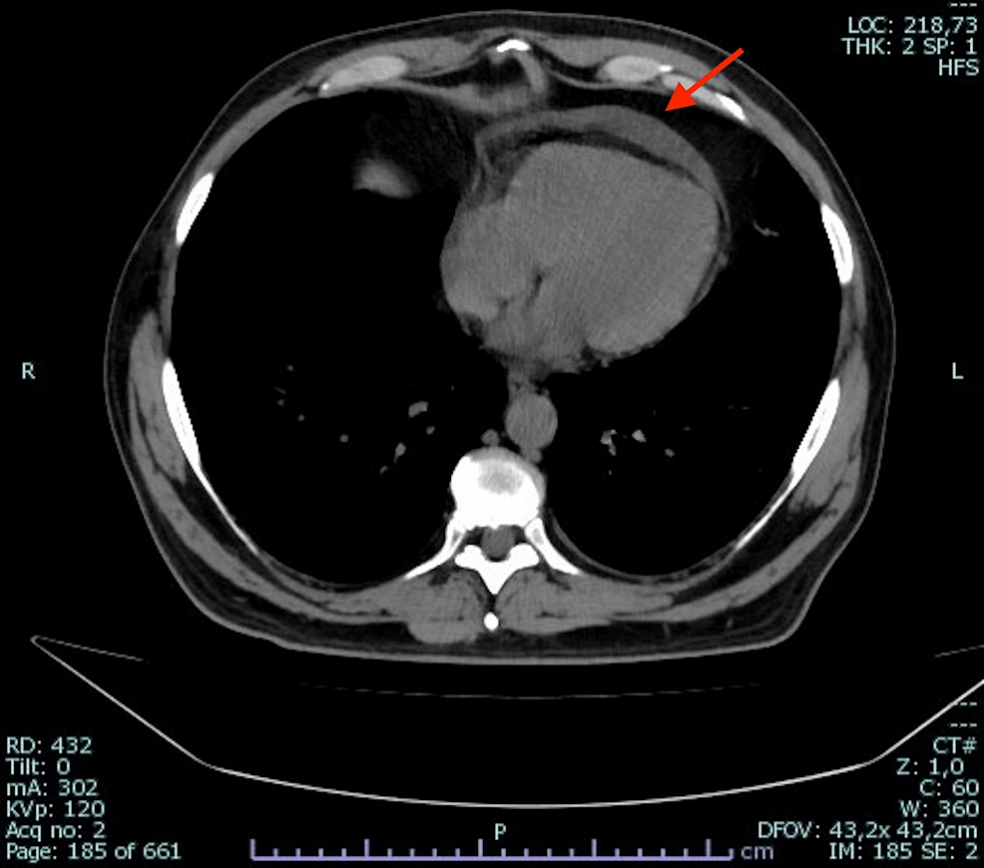
Chest CT shows a pericardial effusion with a diameter of 1.9 cm (arrow).

In August 2022, a 60-year-old man presented to our department because of a fever (up to 39°C) for two months. He complained of chills, headache, dry cough, diffuse arthralgia, and myalgia. He took several oral and intramuscular antibiotics without any improvement. Medical history was positive for a follicular lymphoma treated with obinutuzumab for the last two years (last administration in February 2022), in remission at the case presentation. He reported a paucisymptomatic SARS-CoV-2 infection in April 2022 and a second SARS-CoV-2 infection needing hospitalization a month before this presentation. He was vaccinated for SARS-CoV-2 with one dose of Moderna in March 2022. On admission, he was febrile at 37.9°C; pulse rate was 100 beats per minute, respiratory rate 26 breaths per minute, blood pressure was 100/70 mmHg and oxygen saturation level 96% in room air. Physical examination was unremarkable except for a mild vesicular murmur reduction on the right lung base. Blood work revealed elevated levels of C-reactive protein (CRP 118 mg/l, reference <5), mild anemia (hemoglobin 11.1 g/dl, reference interval 12-18), acute renal injury (creatinine 1.56 mg/dl, reference interval 0.67-1.17), mild immunoglobulin G level reduction (515 mg/dl, reference interval 700-600), and normal white blood cells count (WBC 6,100 cells/μl) and platelets (PLT 303,000/μl). Procalcitonin value was 0.464 μg/l (reference <0.05, unlikely sepsis <0.5). Nasopharyngeal (NP) swab showed a very SARS-CoV-2 low viral load by real-time polymerase chain reaction test, with a cycle threshold value of 31.0 for ORF1a/b gene and 32.0 for Gene E. The result was interpreted as a low-level SARS-CoV-2 persistence. Chest computed tomography (CT) showed a bilateral patchy distribution of areas with increased density, ground-glass opacification and consolidation (Figure 1) associated with an anterior pericardial effusion (0.9 cm) and a mild left pleural effusion. Blood, urine, and sputum cultures did not report any positive results. Nasopharyngeal and rectal swabs for methicillin-resistant Staphylococcus aureus and Klebsiella pneumoniae carbapenemase-producing bacteria colonization, and human immunodeficiency virus, hepatitis C virus, and hepatitis B virus tests were negative. Considering recent COVID-19, piperacillin-tazobactam 4.5 g four times a day was administered for secondary bacterial pneumonia suspicion. Heparin anticoagulant prophylaxis was used. No improvement was observed, and although an escalation antibiotic therapy with carbapenem was administered, the patient remained febrile, and clinical conditions had a progressive deterioration. The bronchoscopy with broncho-alveolar lavage highlighted Pseudomonas aeruginosa and Stenotrophomonas maltophilia growth but targeted antibiotic therapy with ceftolozane-tazobactam and trimethoprim-sulfamethoxazole did not lead to any improvement in the patient. Fungi culture and blood and broncho-alveolar galactomannan were negative. The patient worsened; he became unable to walk unassisted due to generalized weakness, and oxygen therapy via a Venturi mask was necessary. On the 30th day of hospitalization, the fever persisted (39°C), blood exams showed pancytopenia (WBC 2,030 cells/μl, platelets 19,000/μl), high level of CRP (284 mg/l), 6-interleukin (489 pg/ml, reference <7), and ferritin (5, 483 mg/dl, reference interval 30-400). Procalcitonin remained stable in confront of admission value. Total body CT and bone marrow biopsy excluded lymphomas relapse because of absence of lymphadenopathy, and non-suggestive histology. Echocardiography and chest CT showed an increase in the pericardial effusion (1.9 cm, Figure 2), and physical examination became remarkable for non-purulent bilateral conjunctivitis, angular cheilitis, and macular erythematous lesions on the right arm. Due to the exclusion of other diagnoses, and the clinical and laboratory picture, an MIS-A was suspected (Table 1). A single dose of intravenous immunoglobulin (2 g/kg) and methylprednisolone (2 mg/kg) were administered. The therapy determined persistent apyrexia from the day following administration, and it resulted in a progressive improvement of the general conditions, reduction of the inflammation markers and normalization of hemoglobin and blood count values. The patient was discharged without oxygen therapy, and he was able to walk without assistance after one week. SARS-CoV-2 low-level viremia on NP swab persisted at the discharge. He reported a full recovery and no recurrence of fever in the following six months.

**Table 1:** MIS-A case definition and diagnostic criteria according to the Centers for Disease Control and Prevention and the criteria present in our case.

MIS-A case definition	Our case
Patient aged ≥21 years hospitalized for ≥24 hours	Yes
Exclusion of alternative diagnosis	Yes
Fever (≥38.0 C) for ≥24 hours prior to hospitalization or within the first three days of hospitalization	Yes
Clinical criteria	
Severe cardiac illness (myocarditis, pericarditis, coronary artery dilatation/aneurysm, or new-onset right or left ventricular dysfunction, 2nd/3rd degree A-V block, or ventricular tachycardia)	Yes (pericarditis)
Rash and non-purulent conjunctivitis	Yes
Secondary clinical criteria	
New-onset neurologic signs and symptoms (encephalopathy, seizures, meningeal signs, or peripheral neuropathy)	No
Shock or hypotension not attributable to medical therapy	No
Abdominal pain, vomiting, or diarrhea	No
Thrombocytopaenia (platelet count <150,000/μl)	Yes
Laboratory evidence	
Elevated levels of at least TWO of the following: C-reactive protein, ferritin, IL-6, erythrocyte sedimentation rate, procalcitonin	Yes
A positive SARS-CoV-2 test for current or recent infection by RT-PCR, serology, or antigen detection	Yes

## Discussion

MIS-A is a rare condition involving some people with previous or current COVID-19. In our case, the patient had two precedent SARS-CoV-2 infections, the first mild symptomatic and the second after three months needing hospitalization. NP swab also showed low-level SARS-CoV-2 positivity during admission in our department and remained positive surprisingly at the discharge. This low-level persistence is probably related to the immunosuppression due to obinutuzumab, an anti-cd20 monoclonal antibody that acts on B-cells, with an effect that can last until 18 months [6]. The pathophysiology of MIS‐A is poorly understood. It is presumed to be related to an antibody‐mediated process or dysregulated immune activation [3]. We believe that SARS-CoV-2 persistence in our case may have resulted in hyperstimulation of the immune system, triggering the development of MIS-A. We do not exclude that the same viral strain caused the first episode of COVID-19 in April and the two subsequent hospitalizations due to the lack of viral clearance associated with immunosuppression. MIS-A is a very complicated and challenging exclusion diagnosis. Clinical features are heterogeneous and include fever, hematologic, gastrointestinal, cutaneous, and cardiovascular involvement [1,2]. The disease may be life-threatening, causing septic shock-like syndromes [7], which, if unrecognized, is lethal. Differential diagnosis includes Kawasaki disease, tick-born infection, viral gastroenteritis, sepsis, hemorrhagic fever, hemophagocytic lymphohistiocytosis, toxic shock syndrome, macrophage activation syndrome, and several other conditions. In our case, the diagnosis was suspected, after a scrupulous exclusion of other conditions, including Hemophagocytic lymphohistiocytosis and the reactivation of lymphoma. The patient fulfilled the primary clinical criteria because of a pericardial effusion compatible with pericarditis and the appearance of non-purulent conjunctivitis, and a localized skin rash on the right arm. The severe thrombocytopenia met the secondary clinical criteria, and hyperinflammation markers with a positive SARS-CoV-2 test the laboratory criteria (Table 1). Although MIS-A cases associated with lymphadenopathy are described in the literature [7,8], no lymphadenopathy was highlighted in our case, and it helped us exclude the reactivation of lymphoma. Chest CT showed a patchy pneumonia aspect that could be mistaken for viral pneumonia with bacterial superinfection. Considering the non-responsive to the antibiotics administered, we think the lung lesions described were closely related to the inflammation associated with MIS-A. A previous MIS-A case series showed that ten patients had pulmonary ground glass opacities, and six had pleural effusions identified on chest imaging despite minimal respiratory symptoms [9]. It was similar to our case, in which oxygen supplementation was not required at admission, despite the lung pattern. In the same study, three patients had positive SARS-CoV-2 PCR test result a month before admission and continued positive PCR test results at the time of admission, like in our case [9]. According to the treatment, there are no clear indications for MIS-A. Intravenous immunoglobulin and steroids were the most used therapy. Also, tocilizumab, an IL‐6 receptor inhibitor, and anakinra, an IL‐1 receptor antagonist, were occasionally administered [1,2,5,10]. Aspirin has been used due to coronary artery involvement, and anticoagulant prophylaxis is often used for the hypercoagulable state [1]. In our case, we observed a rapid and optimal response to intravenous immunoglobulin administration associated with steroids. The patient had an immediate defervescence, and he remained afebrile.

## Conclusions

MIS-A is a life-threatening condition of unknown pathophysiology associated with previous or current SARS-CoV-2 infection. The disease is characterized by heterogeneous and nonspecific clinical pictures. Diagnosis is challenging, and high suspicion should be maintained in the cases of severe sepsis-like illnesses unresponsive to antimicrobial therapy, where other conditions are excluded. Our case highlighted a presentation of an MIS-A in an immunocompromised patient with SARS-CoV-2 infection persistence. Due to the late diagnosis, the patient worsened, but with steroids and intravenous human immunoglobulin administration, we observed a full recovery. Further case descriptions are needed to identify common guidelines for MIS-A management.
